# Regulation of the microRNA profiles related to Myh7 and Myh6 in myocardial ischemia through proanthocyanidins and different intensity exercise training

**DOI:** 10.22038/AJP.2024.24739

**Published:** 2025

**Authors:** Safar Zarei, Farzaneh Taghian, Gholamreza Sharifi, Hassanali Abedi

**Affiliations:** 1 *Department of Sports Physiology, Faculty of Sports Sciences, Isfahan (Khorasgan) Branch, Islamic Azad University, Isfahan, Iran*; 2 *Research Center for Non-Communicable Diseases, Faculty of Medicine, Jahrom University of Medical Sciences, Jahrom, Iran*

**Keywords:** Proanthocyanidins, High-intensity interval training, Myocardial ischemia, mRNA, microRNAs

## Abstract

**Objective:**

Myocardial ischemia (MI) and circulatory arrest are associated with unfavorable cardiovascular outcomes. This study aims to investigate the effects of proanthocyanidins (PC) and regular exercise with various intensity training protocols (low, moderate, and high) on cardiac protection in a rat model of MI induced by isoproterenol.

**Materials and Methods:**

Based on bioinformatics, a pool of microRNAs and mRNAs was assessed according to significant differential expression in MI condition. Further, the networks of hub genes and mRNA-microRNAs were constructed. After 14 weeks of low, moderate, and high-intensity interval training and oral administration of 300 mg/kg of PC, MI was established in the rats by injecting isoproterenol. The real-time qPCR assessed the relative expressions.

**Results:**

Based on the *in-silico* analysis, *Fn1* (fibronectin-1) and Myh7 (myosin heavy chain 7) are potentially druggable cut points to reduce cardiac tissue damage. High-intensity interval training (HIIT) and consumption of PC modified the relative expression of Myh6 (myosin heavy chain 6), *Myh7*, and *Nf1*. Moreover, High-intensity interval training and PC regulated the mir92a-3p, mir181a-5p, mir29a-3p, and mir133a-3p.

**Conclusion:**

Here, the data indicated that the HIIT protocol could have an effective strategy compared to low-intensity interval training (LIIT) and moderate-intensity interval training protocols (MIIT). Furthermore, HIIT and PC might have protective effects on the MI condition.

## Introduction

Ischemic cardiomyopathy (ICM) is the primary cause of mortality worldwide (Bhandari et al., 2021). Moreover, based on the epidemiological evidence, myocardial ischemia (MI) accounts for around 80% of all cardiovascular diseases (CVDs) (Kurian et al., 2016). Pathogenesis of CVDs has been linked to oxidative stress, inflammation, apoptosis, and disruption in cardiomyocyte contraction elements (Pakravan et al., 2022). 

In animal models, MI and other forms of death in the cardiac tissue can be triggered by isoproterenol, a sympathomimetic beta-adrenergic receptor agonist (Hamilton et al., 2003). 

Medical intervention strategies implemented to optimize, manage, and treat patients with ICM in the hospital, consist of revascularization, aspirin, beta-adrenergic antagonist (beta-blockers), high potency statins, angiotensin-converting enzyme inhibitors, angiotensin II receptor blockers, hydralazine and nitrate, angiotensin receptor neprilysin inhibitor (ARNI), spironolactone, digoxin, inhibitors of the cardiac late sodium current, implantable cardioverter defibrillators (ICD) placement, and biventricular pacing (Bhandari et al., 2021). However, there are effective complementary and alternative medicine strategies for preventing and improving healthcare during pathogenesis statuses (Hajibabaie Abedpoor Safavi et al., 2022). Based on the evidence, exercise, diets, and exogenous antioxidants are complementary strategies to boost the body's natural defenses (Abedpoor et al., 2022b; Akbarian et al., 2021; Hajibabaie et al., 2023). Many fruits include exogenous antioxidants, vitamins C and E, and polyphenols like flavonoids, folic acid, and carotenoids (Gomes et al., 2012; Kurutas, 2015). The red grape seed extract has been proven to be an effective antioxidant in several scientific studies (Habib et al., 2022; Krasteva et al., 2023). Our literature review has indicated that one of the practical biological active components derived from grape seed is proanthocyanidins, which could have positive protective effects as antioxidants of at least 20 and 50 times more than vitamins E and C, respectively (Bagchi et al., 1997; Foshati et al., 2021). In addition, evidence has indicated that PC reduced cell death by increasing the efficiency of the endogenous antioxidant system and decreasing xanthine oxidase levels (Kadri et al., 2021). On the other hand, proanthocyanidins have been found to protect cardiac tissue from ischemia/reperfusion injury and vascular damage by blocking vascular endothelial growth factors, decreasing the angiogenesis process, and controlling the extracellular matrix (Braile et al., 2020). Besides, ample evidence reported that regular physical activity could improve whole-body performance, cardiorespiratory capacity, metabolic features, chronic inflammation, and oxidative stress via cross-talk signaling cascades (Abedpoor et al., 2022b; Haghparast Azad et al., 2022; Rahimi et al., 2021). 

Evidence reported that only two specific myosin heavy chains (MYH) proteins, myosin heavy chain alpha-subunit (*Myh6*) and myosin heavy chain beta-subunit (*Myh7*), are present in mammalian cardiac tissue. The mature heart expresses and distributes these proteins differently than during development. As in other mammals, Myh7 is highly expressed in the ventricles of the heart tissue, while *Myh6* is more predominant in the heart's chambers (Broadwell, 2022). Myh6 encodes cardiac myosin alpha heavy chain components in the developing atria (Broadwell, 2022). It has been observed that mutations of *Myh6* correlate with hypertrophic and dilated cardiomyopathy (Broadwell, 2022). Myh6 was connected with congenital heart disease and implied that increasing mutation of *Myh6* might be associated with congenital heart disease (Razmara et al., 2018). In addition, pathogenesis may be influenced by genetic regulators that modulate gene expression (Abedpoor et al., 2022a; Hajibabaie Abedpoor Assareh et al., 2022; Hajibabaie et al., 2020). MicroRNAs and other non-coding RNAs play an essential role in post-translational regulation and can control gene expression by binding to a specific sequence in the 3'-untranslated region (UTR) of mRNA (Hajibabaie Abedpoor Assareh et al., 2022; Hajibabaie et al., 2020). Extensive research has linked microRNAs to cardiac pathophysiology, suggesting they play crucial roles in atherosclerotic lesion development and ischemia/reperfusion events leading to MI (Hajibabaie et al., 2020). Differential gene expression profiles and potentially pathogenic conditions can result from changes in the expression profiles of microRNAs and the affinities with which they bind to target mRNAs (Hajibabaie et al., 2020). 

Hence, the current study investigated the effects of proanthocyanidins and regular exercise with various intensity training protocols (low, moderate, and high) as cardiac protective factors in a rat model of MI induced by isoproterenol hydrochloride. In this study, we conducted bioinformatics and chemoinformatics analysis and recognized the pivotal genetic interactions network involved in the cardiac pathogenesis status and regulatory molecules.

## Materials and Methods

### Animal grouping, proanthocyanidins bioactive compound consumption, and exercise training protocols

The Jahrom University of Medical Sciences animal house provided 54 male Wistar rats weighing 160-180 g. The rats were housed in a controlled environment with a regular 12-hr light/dark cycle, a temperature of 22±3°C, and humidity of approximately 50-55 %. 

Proanthocyanidins have been purchased from Sigma-Aldrich company (Product No. 1298208, Sigma-Aldrich). After a week of adaptation, the rats were ready for the starting the procedure. Rats exercised with three different intensities of aerobic exercise (low, moderate, and high) for 14 weeks (5 days a week) and gavaged with 300 mg/kg of proanthocyanidins before induction of MI (five days a week). After exercising and consuming proanthocyanidins, MI was induced with isoproterenol hydrochloride (Iso) 24 hr after the last intervention. A total of 9 groups (n=6) of rats were randomly distributed for this study. These groups included:

 (1) Myocardial ischemia rats (MI group), 

(2) Rats without intervention and considered negative control (Normal group), 

(3) Rats treated with proanthocyanidins (PC group), 

(4) Rats that received low-intensity interval training (LIIT group) 

(5) Rats that received the combination PC+low-intensity interval training (PC+LIIT group), 

(6) Rats that received moderate-intensity interval training (MIIT group), 

(7) Rats that received the combination PC+modrate-intensity interval training (PC+MIIT group), 

(8) Rats that received high-intensity interval training (HIIT group), 

and (9) Rats that received the combination PC+high-intensity interval training (PC+HIIT group). 

Notably, the PC (300 mg/kg) was dissolved in 1 ml of normal saline and given orally five days a week for 14 weeks. It should be noted that the dose of proanthocyanidins was selected based on the literature review and data mining (Jhun et al., 2013; Qin et al., 2020).

Research study protocols on animals followed the guidelines established by the Ethics Committee of Jahrom University of Medical Sciences (Ethical code: IR.JUMS.REC.1399.050). 

### Biochemical analysis

This study evaluated the serum concentration of troponin-1 (ZEllBio, RK09281) by commercially available Enzyme-Linked Immunosorbent Assay (ELISA) kits according to the manufacturer's guidelines. 

### Myocardial ischemia induction

Twenty-four hours after 14 weeks of exercise and consuming proanthocyanidins, rats were induced myocardial ischemia/reperfusion by giving an intraperitoneal injection of 80 mg/kg body weight (BW) isoproterenol hydrochloride. The injection was repeated 24 hr later (Frederico et al., 2009). A combination of 50 mg/kg BW ketamine hydrochloride and 10 mg/kg BW xylazine hydrochloride was used to induce anesthesia in the rats 4 hr after the second injection. Heart tissues were quickly frozen in liquefied nitrogen and stored at -80ºC until RNA extraction (Lobo Filho et al., 2011). 

### Intervention protocols of exercise training (low, moderate, and high intensities)

The low-intensity, moderate-intensity, and high-intensity training programs were designed based on the maximum oxygen uptake (VO_2_ max) and intensity. In the first week, the rats ran on treadmills for 15 min at a speed of 5 m/min and a slope of 0 degrees to promote adaption. Each workout included a 5-min warm-up and a 5-min cool-down. The protocol for moderate-intensity interval training was as follows: running speed and VO_2_ max consistently enhanced to reach 30 m/min and 70% VO_2_ max. Also, the slope of the MIIT gradually increased to reach 5 degrees. Moreover, the protocol for high-intensity interval training was as follows: running speed and VO_2_ max consistently increased to reach 35 m/min and 90% VO_2 _max. Furthermore, the slope of the HIIT progressively enhanced to 10 degrees. In addition, the protocol for low-intensity interval training was as follows: running speed and VO_2 _max consistently amplified to reach 20 m/min and 61% VO_2 _max_. _The slope of the LIIT was considered 0 (Abedpoor et al., 2018; Bahadorani et al., 2019).

### Gene expression evaluation (quantitative real-time PCR)

Heart tissues were snap-frozen in liquid nitrogen for RNA extraction and preserved at -80°C. *Fn1*, *Myh6*, and *Myh7* gene expression levels were evaluated by real-time qPCR using the SYBR Green technique. Moreover, total RNAs were extracted from heart tissue using the TRIZOL reagent following the manufacturer's procedure (Invitrogen, Carlsbad, CA). Finally, measurement of extracted RNAs was conducted to analyze the purity and concentration of the RNA samples by NanoDrop spectrophotometer (Thermo Scientific). 

cDNA synthesis was used following the instructions provided by the Fermentas kit (Fermentas, Hanover, MD). The cDNAs were stored at -80°C for the subsequent investigation. Using a real-time PCR cycler and based on the manufacturer's recommended procedure for the kit (TAKARA BIO INC), RT-qPCR using the SYBR Green technique was conducted (Rotor-Gene QIAGEN). Primers were designed in the BEACON primer designer tool and Oligo 7 software, which is mentioned in the primer sequences in the following (forward and reverse): *Fn1*: 5'-CTGGTTACCCTTCCACACCC-3', 5'-GGTGACGAAGGGGGTCTTTT-3', *Myh6* 5'- TCATGCGCATTGAGTTCAAGA-3', 5'-AGTAGAGCTTCATCCACGGC-3', *Myh7* 5'-GGAGAGCATCATGGACCTGG-3', 5'-TCCTGGCGTTGAGTGCATTT-3', *Gapdh*: F: 5'-AGTGCCAGCCTCGTCTCATA-3' and R: 5'-GAGAAGGCAGCCCTGGTAAC-3'. The 2^–ΔΔCT^ statistic was used to analyze the relative expression levels of the appointed genes with reference to the Gapdh housekeeping gene.

### Screening of genetic factors associated with myocardial ischemia-reperfusion injury

The systematic bioinformatic survey indicated the significant differential hub genes in the MI status. This evidence was obtained from transcriptomic microarray datasets and showed the expression profile of genes. The advanced analysis detected genes with significant differential expression in the pathogenesis compared with normal conditions, and strong molecular techniques should confirm these gene expression profiles. Based on bioinformatic analysis, this study determined several hub genes with the highest degree and betweenness centrality, and qRT-PCR assessed their expression profile. Hence, we noticed the hub genes associated with MI pathogenesis, which might be introduced as monitoring biomarkers. To obtain the list of the genes related to MI, an expression profiling array with GSE ID: 160516 was browsed in the Gene Expression Omnibus (GEO) database, and bioinformatics analysis to uncover hub genes implicated in MI by R programming language for statistical computing and graphics. In this study, we considered differential expression of genes with p<0.05 and logarithm Fold Change (log FC) cut-off ±0.03 to significantly highlight genes with down and up-regulation patterns. Based on the Panther database and Kyoto Encyclopedia of Genes and Genomes (KEGG) we explored the pathways involved in the MI status. Based on bioinformatics analysis, *Fn1*, *Myh6*, and *Myh7* were selected for the experimental assay. On the other hand, we reviewed the miRWalk databaseand literature to predict possible microRNAs that target selected candidate genes. Finally, the interconnections are plotted between genes, microRNAs, and MI status in a comprehensive network.

### Virtual screening of Proanthocyanidins

The *in-silico* study found that Fn1 acted as a mediator between oxidative stress agents and cardiac cellular contraction, regulating the expression of genes and proteins implicated in cardiac pathogenicity. Consequently, a pharmaceutical design approach was proposed that considers the structure of the Fn1 and Myh7 proteins based on molecular docking prediction to improve cardiac tissue in myocardial ischemia-reperfusion injury by employing proanthocyanidins as a preventive compound. Three-dimensional (3D) structures of Fn1 (ID: 3m7p) and Myh7 (ID: 4db1) proteins based on the X-ray diffraction method were browsed in the Protein Data Bank server. Proanthocyanidins' three-dimensional structure from the PubChem database was exported in the SDF format. Moreover, the structures of the Fn1 and Myh7 proteins were prepared and optimized in UCSF Chimera 1.8.1. PyRx software was used to predict the binding affinity of proanthocyanidins to macromolecules (*Fn1* and *Myh7*) in the search box.

### Statistical calculating

GraphPad Prism was used for the statistical analysis of variance (Version 9 Graph Pad Software Inc., La Jolla, CA). The Kolmogorov-Smirnov test was performed for normalizing. Due to the need to make comparisons across groups, the one-way analysis of variance (ANOVA) was used to examine the data. All analyses considered differences at the p.value <0.05 level significant. Data are displayed as mean and standard deviation (SD).

## Results

### Hub genes, regulatory factors, and molecular-cellular signaling pathways involved in the myocardial ischemia-reperfusion injury based on bioinformatics prediction

**Figure 1:**
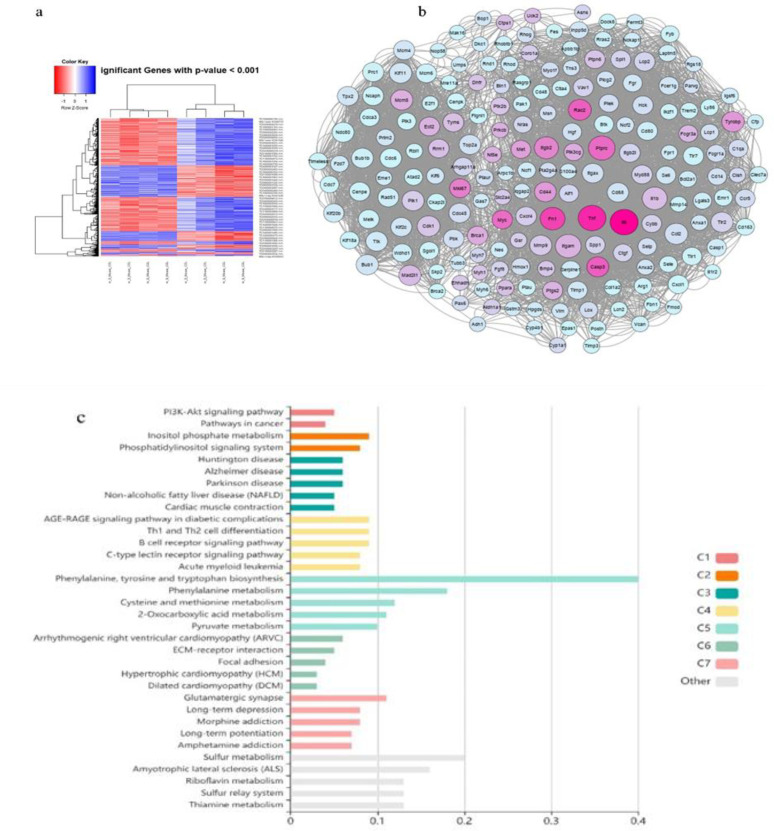
a: The heatmap diagram demonstrates the significant genes with differential expression in the myocardial ischemia-reperfusion injury vs. sham group (p<0.001). b: The biological protein-protein interactions network marked 195 significant hub genes with pathogenic expression levels that could be involved in the pathophysiology of myocardial ischemia-reperfusion injury. c: The significant molecular signaling pathways specified complications in the myocardial ischemia-reperfusion injury pathogenesis based on enrichment analysis.

Among the 6127 genes with significantly differential expression in the R analysis of the myocardial ischemia-reperfusion injury dataset, 3007 were overexpressed, and 3080 were downregulated considering the p<0.05 threshold and log FC±0.03 cut-off. The heat map diagram displays significant genes with differential expression in the myocardial ischemia-reperfusion injury compared to the control samples (p<0.001) (Figure 1a). The protein-protein interactions (PPIs) network consists of significant genes constructed by visualizing network parameters such as betweenness centrality, closeness centrality, and degree in the CytoScape software. This PPIs network marked significant hub genes (195 hub genes) that could be involved in the pathophysiology of myocardial ischemia (Figure 1b). The enrichment and text mining of significant hub genes have shown that these genes are involved in pathological cardiac ischemia-reperfusion damage conditions via molecular signaling pathways (Figure 1c and Table 1).

**Figure 2:**
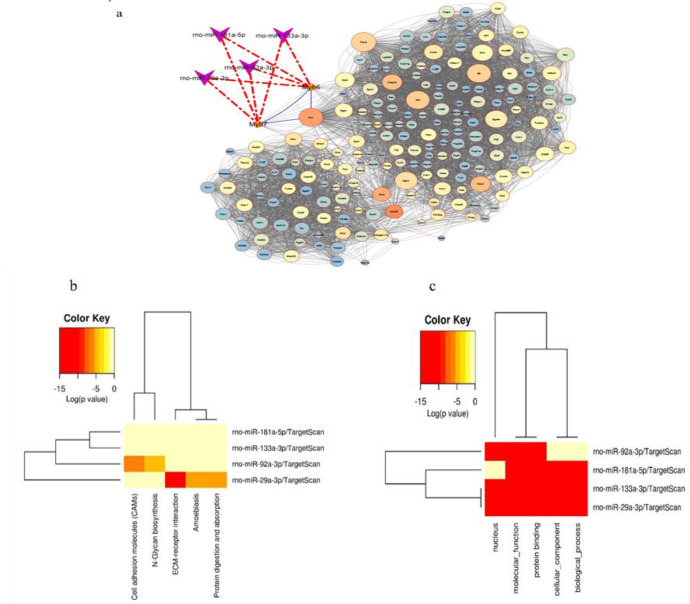
. a: Construction of a genetics network of potential microRNAs targeting significant genes involved in cardiac dysfunction after myocardial ischemia-reperfusion injury. b and c: The signaling pathways related to appointed microRNAs are shown as a heatmap graph based on Kyoto Encyclopedia of Genes and Genomes (KEGG) and gene ontology (GO) analysis using a p-value of 0.05, Score type context +, and Fisher's exact test enrichment algorithms, respectively.

Prediction of microRNAs for target genes indicated that miR-29a-3p, miR-92a-3p, miR-133a-3p target Myh6 in the CoDing Sequence (CDS) region, and miR-133a-3p, miR-92a-3p, miR-181a-5p, and miR-29a-3p target Myh7 in the CDS region. We constructed a network to map out potential repair mechanisms based on our knowledge of the genes and microRNAs involved in cardiac dysfunction (Figure 2a). We postulated that alterations in gene expression and microRNAs might impair normal activities of cardiac tissue and result in heart injury by interfering with crucial signaling networks. Moreover, the miRPath v.3 servers were used to analyze gene ontology data and data from the Kyoto Encyclopedia of Genes and Genomes to display the significant associations between signaling pathways and microRNAs (Figures 2b and c).

### Virtual screening results.

**Figure 3:**
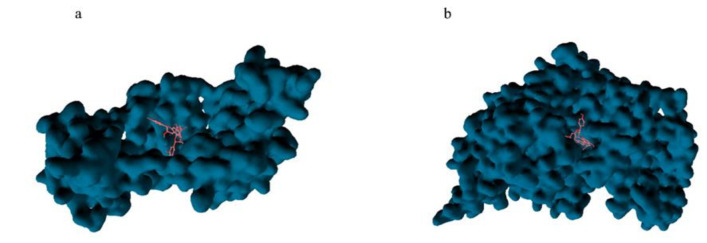
a and b: Molecular docking simulations predicted an optimal binding affinities score <-5 kcal/mol and RMSD<2 for a potential interaction between proanthocyanins and the FN1 and Myh7 proteins, respectively, during a virtual screening.

Using a molecular docking approach, we calculated the binding affinity between a small molecule, proanthocyanidins, and the main chain of Fn1 and MYH7 proteins, predicting a suitable docking score (binding affinity <-5 kcal/mol and RMSD<2). Figures 3a and 3b display the optimal binding affinities for Fn1: Proanthocyanidins (-8.6 kcal/mol) and MYH7: Proanthocyanidins (-10.1 kcal/mol).

### Cardiac troponins I (TnI) improved by high-intensity interval training and consumption of proanthocyanidins

**Figure 4:**
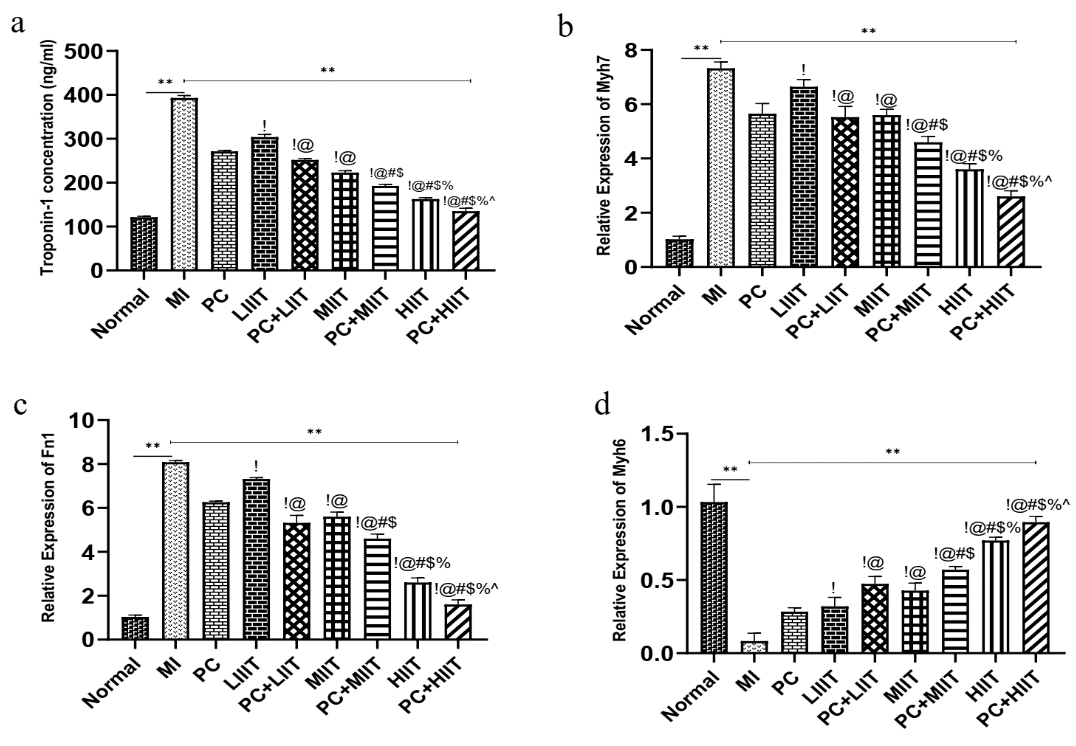
The concertation of the troponin I and expression level of the *Myh7*, *Nf1*, and *Myh6*. a. The concertation of the troponin I (ng/ml), b. The relative expression of the *Myh7*, c. The relative expression of the *Fn1*, d. The relative expression of the *Myh6*. !: Demonstrates a statistically significant difference with the PC group at p <0.05, @: Demonstrates a statistically significant difference with the LIIT group at p<0.05, #: Demonstrates a statistically significant difference with the PC+LIIT group at p<0.05, $: Demonstrates a statistically significant difference with the MIIT group at p<0.05, %: Demonstrates a statistically significant difference with the PC+MIIT group at p<0.05. ^: Demonstrates a statistically significant difference with the HIIT group at p<0.05.

The cardiac troponin I (TnI) concentration was assayed to determine inducing myocardial ischemia-reperfusion injury in the rat model (Figure 4a). The data indicated that the concentration of TnI in the myocardial ischemia rat model significantly increased compared with the normal group (Figure 4a). Moreover, we indicated that the concentration of the TnI was reduced via consumption of the Proanthocyanidins and exercise training in different intensities (low, moderate, and high-intensity interval training) compared with the MI group (Figure 4a). Furthermore, the combination PC+low-intensity interval training (PC+LIIT), combination PC+modrate-intensity interval training (PC+MIIT), and combination PC+high-intensity interval training (PC+HIIT) significantly decreased the concentration of the TnI in comparison to MI, PC, LIIT, MIIT, and HIIT groups (Figure 4a). In addition, results demonstrated that the concentration of the TnI declined compared with the other groups. Hence, the data revealed that PC+HIIT improved myocardial ischemia (Figure 4a).

### High-intensity interval training and consumption of proanthocyanidins modified the relative expression of the Myh6, Myh7, and Nf1

Enhanced Myh7 and Nf1 were found in the heart of myocardial ischemia rat model in compression with the normal group (Figures 4b, c). Moreover, the data indicated that consumption of Proanthocyanidins significantly decreased the expression level of Myh7 and Nf1 (Figures 4b and c). Furthermore, data demonstrated that different exercise training intensities reduced the *Myh7* and *Nf1* genes (Figures 4b, c). Based on these data, low-, moderate-, and high-intensity interval training could regulate the expression level of the *Myh7* and *Nf1* (Figures 4b and c). In addition, our results indicated that the combination PC+low-intensity interval training (PC+LIIT), combination PC+modrate-intensity interval training (PC+MIIT), and combination PC+high-intensity interval training (PC+HIIT) predominantly reduced the relative expression of Myh7 and Nf1 *Vs. *MI, PC, LIIT, MIIT, and HIIT (Figures 4b, c). Interestingly, the *Myh7* and *Nf1* expression levels significantly decreased compared with the other groups. Hence, the data demonstrated that PC+HIIT was more favorable than other interventions (Figures 4b and c). 

On the other hand, the expression level of the Myh6 declined in the MI rats compared with the normal group (Figure 4c). Furthermore, PC enhanced the expression level of the Myh6 compared with the MI group (Figure 4c). Moreover, low-, moderate-, and high-intensity interval training modified the relative expression of the Myh6 (Figure 4c). Notably, high-intensity interval training remarkably elevated the expression of the Myh6 in compression to low- and moderate-intensity interval training (Figures 4c). Moreover, the combination of PC+high-intensity interval training (PC+HIIT) *Vs. *other groups significantly upregulated the expression of the Myh6 (Figure 4c).

### High-intensity interval training and consumption of Proanthocyanidins regulated the mir92a-3p, mir181a-5p, mir29a-3p, and mir133a-3p

**Figure 5:**
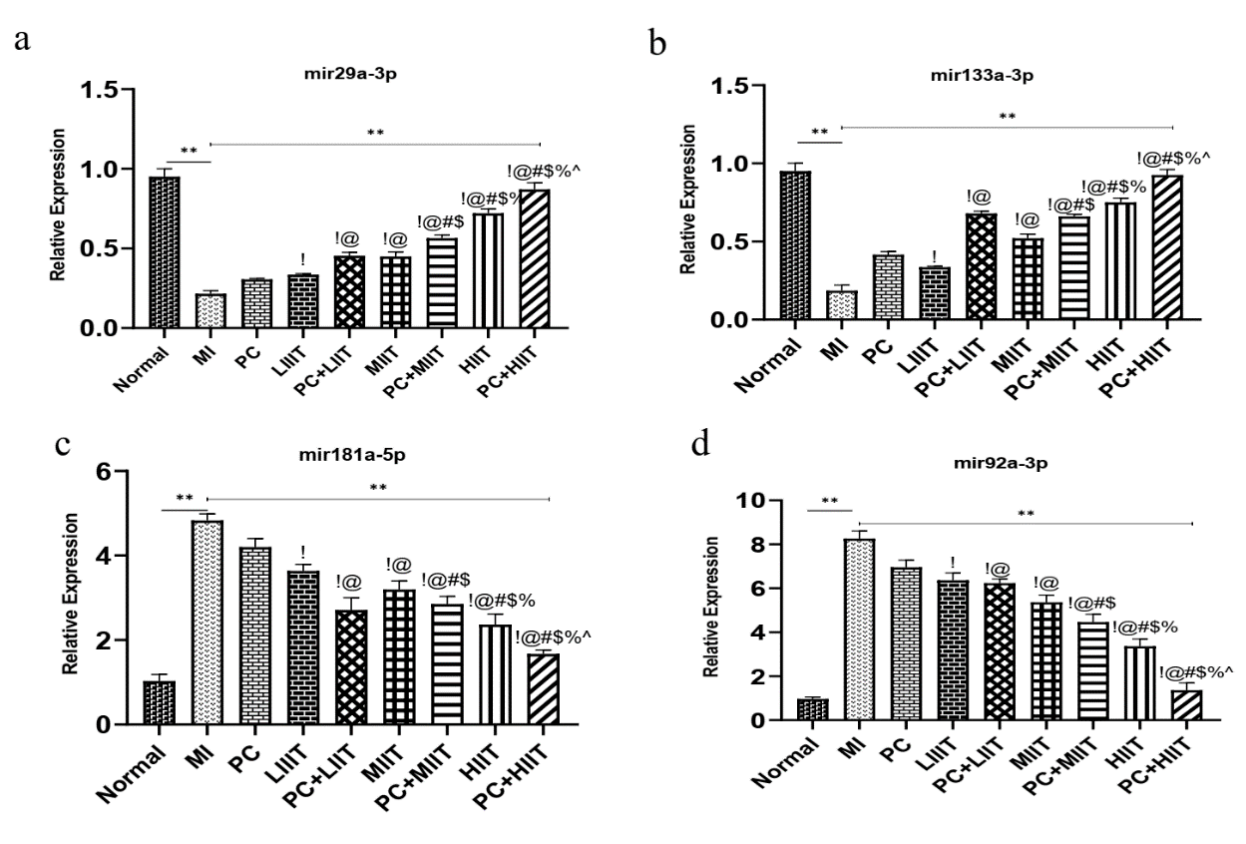
Expression level of miR-29a-3p, miR-133a-3p, miR-92a-3p, and miR-181a-5p. a. The relative expression of miR-29a-3p, b. The relative expression of the miR-133a-3p, c. The relative expression of the miR-181a-5p, d. The relative expression of the miR-92a-3p. !: Demonstrates a statistically significant difference with the PC group at p <0.05, @: Demonstrates a statistically significant difference with the LIIT group at p<0.05, #: Demonstrates a statistically significant difference with the PC+LIIT group at p<0.05, $: Demonstrates a statistically significant difference with the MIIT group at p<0.05, %: Demonstrates a statistically significant difference with the PC+MIIT group at p<0.05. ^: Demonstrates a statistically significant difference with the HIIT group at p<0.05.

The expression level of the miR-29a-3p and miR-133a-3p significantly decreased in the MI group (Figures 5a, b). Hence, reducing the miR-29a-3p and miR-133a-3p led to overexpression of the Myh7 and Nf1 genes in MI conditions (Figures 4b and c). Moreover, data indicated that Proanthocyanidins, LIIT, MIIT, and MIIT, could modify the expression level of the miR-29a-3p and miR-133a-3p (Figures 5a and b). Besides, the combinations of PC+low-intensity interval training (PC+LIIT), combination PC+modrate-intensity interval training (PC+MIIT), and combination PC+high-intensity interval training (PC+HIIT) significantly upregulated the expression of miR-29a-3p and miR-133a-3p (Figures 5a and b). In addition, data revealed that PC+HIIT predominantly enhanced the expression of miR-29a-3p and miR-133a-3p compared with the other groups (Figures 5a, b).

On the other hand, the expression level of the miR-92a-3p and miR-181a-5p, which could target the Myh6 genes, significantly increased in MI conditions compared with Normal (Figures 5c and d). Furthermore, the Proanthocyanidins and exercise training in different intensity programs (low-, moderate-, and high-intensity interval training) decreased the expression level of the miR-92a-3p and miR-181a-5p compared with the MI group (Figures 5c, d). Moreover, high-intensity interval training reduced the expression level of these miRNAs compared with the PC, LIIT, and MIIT groups (Figures 5c and d). Interestingly, combining the Proanthocyanidins and exercise training in different intensity programs reduced the expression level of the miR-92a-3p and miR-181a-5p. Notably, data determined that the combination of PC+high-intensity interval training (PC+HIIT) downregulated the expression of the miR-92a-3p and miR-181a-5p compared with the other groups (Figures 5c and d). 

## Discussion

This study sought to determine the association between candidate miRNAs and hub genes in MI conditions. We evaluated the different intensity exercise training and consumption of the Proanthocyanidins on alteration of the selected hub genes and miRNAs in myocardial ischemia-reperfusion injury rats. 

Myocardial dysfunctions were linked to specific molecular signaling pathways. Based on the bioinformatics analysis, *Myh6, Myh7, *and* Fn1* were selected to have pivotal roles in cardiac cell contraction and ischemic cardiomyopathy. A previous study by Chen *et al.* revealed that myh6 is the pivotal hub gene involved in myocardial ischemia pathogenesis with lower expression levels in coronary artery disease, heart failure, and acute MI (Chen et al., 2021).

On the other hand, Broadwell attributed the contractile function of cardiac muscle to the expression balance of *Myh6/Myh7* (10%:90%) and showed that this ratio could be crucial in cardiac dysfunction (Broadwell, 2022). In this study, our data indicated that the expression level of *Myh6* was decreased, and the *Myh7* expression level was upregulated in the MI.

Moreover, the relative expression of *Fn1* in the MI group was elevated compared to the normal group. Immense evidence has indicated that Fn1 expression is upregulated in MI. Furthermore, Fn1 polymerization is needed for collagen sediment and plays a vital position in myocardial ischemia/reperfusion injury-induced inflammation, myocardial fibrosis, and neovascular formation after infarction (Konstandin et al., 2013; Valiente-Alandi et al., 2018). 

The medical treatment of cardiovascular illnesses may benefit from complementary medicine and commercial/conventional pharmaceutical medication. The most common forms of complementary medicine are herbal remedies and regular physical activity (Adib-Hajbaghery et al., 2021). Grape seed oil is commonly believed to be a healthy diet due to its presumed anti-cancer, anti-inflammatory, anti-thrombotic, anti-diabetic, and cardioprotective properties (Habib et al., 2022). Growing evidence has indicated that grape seed compounds could regulate several pathomechanisms, such as oxidative stress, inflammation, *PI3K/Akt* signaling pathway, and cardiac muscle contraction (Sochorova et al., 2020). Oueslati *et al.* found that high-dosage grape seed and skin extract (GSE; 4 g/kg) is a safe and effective antioxidant for the treatment of diabetes complications by reducing oxidative stress and renal dysfunction in diabetic rats (Oueslati et al., 2016). Ruan *et al.* found that consuming proanthocyanidins reduced histological hallmarks of myocardial ischemia in MI mice (Ruan et al., 2020). They also found that proanthocyanidin protected the heart from hypoxia by preventing apoptosis and reducing the expression of the *PI3K-AKT* pathway (Ruan et al., 2020). Shao et al. reported that proanthocyanidin bioactive compound consumption inhibited the relative expression of miR-9, a miRNA that targets ACAT1 (Shao et al., 2020). Proanthocyanidins probably reduced ACAT1 expression by increasing miR-9 expression, reducing intracellular lipid accumulation, and blocking the production of macrophage foam cells. MiR-9 mimic and its inhibitor further confirmed this hypothesis (Shao et al., 2020). Several miRNAs can control cholesterol efflux in macrophages, including miR-33, miR-19b, miR-144, etc., by directly targeting ABCA1 or both ABCA1 and ABCG1. As a result, macrophage foam cell development may be controlled by applying miRNA to the appropriate target (Hajibabaie et al., 2020; Lv et al., 2014; Ouimet et al., 2015).

Hence, in this study, the data indicated that consumption of the proanthocyanidins compound significantly regulated the expression level of the *Myh7, Nf1*, and *Myh6* hub genes and their candidate miRNAs. Furthermore, the data demonstrated that miR-29a-3p, miR-133a-3p, miR-92a-3p, and miR-181a-5p could be biomarkers and regulators for rat cardiac injury. *In-silico* data analysis revealed that miR-29a-3p and miR-133a-3p could bind the Myh7 gene, and miR-92a-3p and miR-181a-5p could target the Myh6 gene. 

We evaluated *Myh7, Myh6, *and *Nf1* expression levels as candidate genes in MI. Moreover, the data indicated that the expression level of miR-29a-3p and miR-133a-3p decreased, and the expression of miR-92a-3p and miR-181a-5p increased in the MI condition. Besides, based on our result, the combination of PC+high-intensity interval training (PC+HIIT) enhanced the expression of the miR-92a-3p and miR-181a-5p targeted the Myh6 gene compared to other groups. Furthermore, data demonstrated that high-intensity interval training along with Proanthocyanidins could downregulate the miR-29a-3p and miR-133a-3p.

Exercise training with various protocols could prevent and minimize heart tissue damage during ischemia. Based on the literature review and data mining, the different intensities of exercise training might be an affordable and practical approach to managing and halting MI. Therefore, increasing exercise intensity and maximizing Vo_2 _max may protect against MI. In addition, several studies have indicated that miRNAs could be crucial mediators of processes associated with exercise training adaption, including hypertrophies, angiogenesis, and cardiac muscle atherosclerosis (Fernandes et al., 2015). Therefore, exercise was hypothesized to protect cardiac tissue by reprogramming miRNAs, genes, and regulatory genetic factors. Data revealed that exercise training with the various intensity training protocols (low, moderate, and high) could regulate the expression level of *Myh7*, *Nf1*, and *Myh6* genes and their candidate miRNAs (miR-29a-3p and miR-133a-3p bound to the *Myh7* and *Nf1* genes and also miR-92a-3p and miR-181a-5p targeted the Myh6 gene) in MI rats.

Interestingly, results demonstrated that among these different protocols, high-intensity interval training was more effective on the expression of miR-29a-3p, miR-133a-3p, miR-92a-3p, and miR-181a-5p in the MI condition. Exercise training has enhanced cardiac function, boosted oxidative phosphorylation, induced reactive oxygen species (ROS) detoxification, and increased mitochondrial size, number, and clearance. Thus, it was hypothesized that consistent training might preserve cardiac tissue by altering the expression of genes and other genetic variables (French et al., 2008; Wang et al., 2015). Nevertheless, reperfusion-induced restoration of oxidative phosphorylation and regeneration of mitochondrial membranes can trigger a cascade of ROS, necrotic cell death, and calcium excess in cardiac cells (Joiner et al., 2012). Fathi investigated the effect of 14 weeks of endurance training (30m/min, 50 min /session) on the expression of the myh6 gene in the left ventricular of rats. According to the results of this study, endurance exercise training, along with structural and functional modifications in the left ventricle, yields differences in the gene level and thereby increases the contractility of the heart (Fathi, 2016). 

On the other hand, data demonstrated that the interactions between proanthocyanidins compounds and exercise training in different intensities greatly affected the expression level of the selected hub genes and miRNAs. The data designated that the combination of PC+low-intensity interval training (PC+LIIT), the combination of PC+modrate-intensity interval training (PC+MIIT), and the combination of PC+high-intensity interval training (PC+HIIT) modified and regulated the relative expression of the *Myh7*, *Nf1*, and *Myh6* genes and their candidate miRNAs (mir29a-3p and mir133a-3p bound to the *Myh7* and *Nf1* genes and mir92a-3p and mir181a-5p targeted the *Myh6* gene) in MI rats. Based on these data, we could conclude that the PC interaction with three types of exercises (low, moderate, and high-intensity interval training) had a synergic effect compared with the other groups. 

Overall, the data indicated that proanthocyanidins consumption as a bioactive compound might significantly protect myocardial dysfunction after myocardial ischemia-reperfusion injury induced by isoproterenol hydrochloride and offset pathological hallmarks of MI. Moreover, exercise has a preventive impact on myocardial ischemia-reperfusion injury by reprogramming genes and genetic regulator factors. 

**Table 1:** Enrichment analysis of hub genes in Panther database

**Term**	**p-Value**	**Corrected p-Value**
Inflammation mediated by chemokine and cytokine signaling pathway	0.00000000000108	0.00000000000454
Integrin signaling pathway	0.000000439	0.00000921
T cell activation	0.00000315	0.0000442
Cytoskeletal regulation by Rho GTPase	0.00000141	0.0000148
Toll receptor signaling pathway	0.0000474	0.000399
Axon guidance mediated by semaphorins	0.000107	0.000749
Huntington disease	0.000199	0.001149
B cell activation	0.000219	0.001149
Ras Pathway	0.000317	0.00133
Interleukin signaling pathway	0.000317	0.00133
p53 pathway feedback loops 2	0.001251	0.004777
FGF signaling pathway	0.001435	0.005024
EGF receptor signaling pathway	0.001569	0.005069
Axon guidance mediated by Slit/Robo	0.001839	0.005518
VEGF signaling pathway	0.00271	0.007587
Angiogenesis	0.004881	0.012814
Axon guidance mediated by netrin	0.007403	0.01829
Apoptosis signaling pathway	0.014467	0.033755
PDGF signaling pathway	0.016811	0.037161
p53 pathway	0.040564	0.077691
Nicotinic acetylcholine receptor signaling pathway	0.04615	0.077691
Endothelin signaling pathway	0.04615	0.077691
Plasminogen activating cascade	0.046245	0.077691
De novo pyrimidine ribonucleotides biosynthesis	0.046245	0.077691
De novo pyrimidine deoxyribonucleotide biosynthesis	0.046245	0.077691
JAK/STAT signaling pathway	0.05747	0.092836
Parkinson disease	0.061236	0.095255
5-Hydroxytryptamine degradation	0.074061	0.111091
Wnt signaling pathway	0.079843	0.115634
Alzheimer disease-presenilin pathway	0.09363	0.129319
Heterotrimeric G-protein signaling pathway-Gq alpha and Go alpha mediated pathway	0.09545	0.129319
Hypoxia response via HIF activation	0.10107	0.132654
Interferon-gamma signaling pathway	0.111653	0.137924
Blood coagulation	0.111653	0.137924
FAS signaling pathway	0.116897	0.138613
p38 MAPK pathway	0.122111	0.138613
DNA replication	0.122111	0.138613
Insulin/IGF pathway-protein kinase B signaling cascade	0.157762	0.174368
Oxidative stress response	0.16768	0.180579
PI3 kinase pathway	0.206207	0.216517
TGF-beta signaling pathway	0.311459	0.319056
Heterotrimeric G-protein signaling pathway-Gi alpha and Gs alpha mediated pathway	0.494266	0.494266

## References

[1] Abedpoor N, Taghian F, Ghaedi K, Niktab I, Safaeinejad Z, Rabiee F, Tanhaei S, Nasr-Esfahani MH (2018). Pparγ/pgc-1α-fndc5 pathway up-regulation in gastrocnemius and heart muscle of exercised, branched chain amino acid diet fed mice. Nutr Metab.

[2] Abedpoor N, Taghian F, Hajibabaie F (2022a). Cross brain–gut analysis highlighted hub genes and lncrna networks differentially modified during leucine consumption and endurance exercise in mice with depression-like behaviors. Mol Neurobiol.

[3] Abedpoor N, Taghian F, Hajibabaie F (2022b). Physical activity ameliorates the function of organs via adipose tissue in metabolic diseases. Acta Histochem.

[4] Adib-Hajbaghery M, Ardakani MF, Sotoudeh A, Asadian A (2021). Prevalence of complementary and alternative medicine (cam) among diabetic patients in eastern mediterranean country members of the world health organization (who): A review. J Herb Med.

[5] Akbarian F, Rahmani M, Tavalaee M, Abedpoor N, Taki M, Ghaedi K, Nasr-Esfahani MH (2021). Effect of different high-fat and advanced glycation end-products diets in obesity and diabetes-prone c57bl/6 mice on sperm function. Int J Fertil Steril.

[6] Bagchi D, Garg A, Krohn R, Bagchi M, Tran M, Stohs S (1997). Oxygen free radical scavenging abilities of vitamins c and e, and a grape seed proanthocyanidin extract in vitro. Res Commun Mol Pathol Pharmacol.

[7] Bahadorani M, Tavalaee M, Abedpoor N, Ghaedi K, Nazem MN, Nasr‐Esfahani MH (2019). Effects of branched‐chain amino acid supplementation and/or aerobic exercise on mouse sperm quality and testosterone production. Andrologia.

[8] Bhandari B, Rodriguez BSQ, Masood W (2021).

[9] Braile M, Marcella S, Cristinziano L, Galdiero MR, Modestino L, Ferrara AL, Varricchi G, Marone G, Loffredo S (2020). Vegf-a in cardiomyocytes and heart diseases. Int J Mol Sci.

[11] Chen JH, Wang LL, Tao L, Qi B, Wang Y, Guo YJ, Miao L (2021). Identification of myh6 as the potential gene for human ischaemic cardiomyopathy. J Cell Mol Med.

[12] Fathi M (2016). The effect of endurance exercise on myh6 gene expression and structural and functional changes of left ventricular. Qom Univ Med Sci J.

[13] Fernandes T, Baraúna VG, Negrão CE, Phillips MI, Oliveira EM (2015). Aerobic exercise training promotes physiological cardiac remodeling involving a set of micrornas. Am J Physiol Heart Circ Physiol.

[14] Foshati S, Rouhani MH, Amani R (2021). The effect of grape seed extract supplementation on oxidative stress and inflammation: A systematic review and meta‐analysis of controlled trials. Int J Clin Pract.

[15] Frederico MJ, Justo SL, Da Luz G, Da Silva S, Medeiros C, Barbosa VA, Silva LA, Boeck CR, De Pinho RA, De Souza CT (2009). Exercise training provides cardioprotection via a reduction in reactive oxygen species in rats submitted to myocardial infarction induced by isoproterenol. Free Radic Res.

[16] French JP, Hamilton KL, Quindry JC, Lee Y, Upchurch PA, Powers SK (2008). Exercise‐induced protection against myocardial apoptosis and necrosis: Mnsod, calcium‐handling proteins, and calpain. FASEB J.

[17] Gomes EC, Silva AN, Oliveira MRd (2012). Oxidants, antioxidants, and the beneficial roles of exercise-induced production of reactive species. Oxid Med Cell Longev.

[18] Habib HM, El Fakharany EM, Kheadr E, Ibrahim WH (2022). Grape seed proanthocyanidin extract inhibits DNA and protein damage and labile iron, enzyme, and cancer cell activities. Sci Rep.

[19] Haghparast Azad M, Niktab I, Dastjerdi S, Abedpoor N, Rahimi G, Safaeinejad Z, Peymani M, Forootan FS, Asadi-Shekaari M, Nasr Esfahani MH (2022). The combination of endurance exercise and sgtc (salvia–ginseng–trigonella–cinnamon) ameliorate mitochondrial markers’ overexpression with sufficient atp production in the skeletal muscle of mice fed ages-rich high-fat diet. Nutr Metab.

[20] Hajibabaie F, Abedpoor N, Assareh N, Tabatabaiefar MA, Shariati L, Zarrabi A (2022). The importance of snps at mirna binding sites as biomarkers of gastric and colorectal cancers: A systematic review. J Pers Med.

[21] Hajibabaie F, Abedpoor N, Safavi K, Taghian F (2022). Natural remedies medicine derived from flaxseed (secoisolariciresinol diglucoside, lignans, and α‐linolenic acid) improve network targeting efficiency of diabetic heart conditions based on computational chemistry techniques and pharmacophore modeling. J Food Biochem.

[22] Hajibabaie F, Abedpoor N, Taghian F, Safavi K (2023). A cocktail of polyherbal bioactive compounds and regular mobility training as senolytic approaches in age-dependent alzheimer’s: The in silico analysis, lifestyle intervention in old age. J Mol Neurosci.

[23] Hajibabaie F, Kouhpayeh S, Mirian M, Rahimmanesh I, Boshtam M, Sadeghian L, Gheibi A, Khanahmad H, Shariati L (2020). Micrornas as the actors in the atherosclerosis scenario. J Physiol Biochem.

[24] Hamilton KL, Staib JL, Phillips T, Hess A, Lennon SL, Powers SK (2003). Exercise antioxidants, and hsp72: Protection against myocardial ischemia/reperfusion. Free Radic Biol Med.

[25] Jhun JY, Moon SJ, Yoon BY, Byun JK, Kim EK, Yang EJ, Ju JH, Hong YS, Min JK, Park SH (2013). Grape seed proanthocyanidin extract–mediated regulation of stat3 proteins contributes to treg differentiation and attenuates inflammation in a murine model of obesity-associated arthritis. PloS One.

[26] Joiner MlA, Koval OM, Li J, He BJ, Allamargot C, Gao Z, Luczak ED, Hall DD, Fink BD, Chen B (2012). Camkii determines mitochondrial stress responses in heart. Nature.

[27] Kadri S, El Ayed M, Kadri A, Limam F, Aouani E, Mokni M (2021). Protective effect of grape seed extract and orlistat co-treatment against stroke: Effect on oxidative stress and energy failure. Biomed Pharmacother.

[28] Konstandin MH, Toko H, Gastelum GM, Quijada P, De La Torre A, Quintana M, Collins B, Din S, Avitabile D, Völkers M (2013). Fibronectin is essential for reparative cardiac progenitor cell response after myocardial infarction. Circ Res.

[29] Krasteva D, Ivanov Y, Chengolova Z, Godjevargova T (2023). Antimicrobial potential, antioxidant activity, and phenolic content of grape seed extracts from four grape varieties. Microorganisms.

[30] Kurian GA, Rajagopal R, Vedantham S, Rajesh M (2016). The role of oxidative stress in myocardial ischemia and reperfusion injury and remodeling: Revisited. Oxid Med Cell Longev.

[31] Kurutas EB (2015). The importance of antioxidants which play the role in cellular response against oxidative/nitrosative stress: Current state. Nutr J.

[32] Lobo Filho HG, Ferreira NL, Sousa RBd, Carvalho ERd, Lobo PLD, Lobo Filho JG (2011). Experimental model of myocardial infarction induced by isoproterenol in rats. BJCVS.

[33] Lv YC, Tang YY, Peng J, Zhao GJ, Yang J, Yao F, Ouyang XP, He PP, Xie W, Tan YL (2014). Microrna-19b promotes macrophage cholesterol accumulation and aortic atherosclerosis by targeting atp-binding cassette transporter a1. Atheroscler.

[34] Oueslati N, Charradi K, Bedhiafi T, Limam F, Aouani E (2016). Protective effect of grape seed and skin extract against diabetes-induced oxidative stress and renal dysfunction in virgin and pregnant rat. BPJ.

[35] Ouimet M, Ediriweera HN, Gundra UM, Sheedy FJ, Ramkhelawon B, Hutchison SB, Rinehold K, van Solingen C, Fullerton MD, Cecchini K (2015). Microrna-33–dependent regulation of macrophage metabolism directs immune cell polarization in atherosclerosis. JCI.

[36] Pakravan G, Peymani M, Abedpoor N, Safaeinejad Z, Yadegari M, Derakhshan M, Nasr Esfahani MH, Ghaedi K (2022). Antiapoptotic and anti‐inflammatory effects of pparγ agonist, pioglitazone, reversed dox‐induced cardiotoxicity through mediating of mir‐130a downregulation in c57bl/6 mice. J Biochem Mol Toxicol.

[37] Qin F, Dong Y, Wang S, Xu M, Wang Z, Qu C, Yang Y, Zhao J (2020). Maximum oxygen consumption and quantification of exercise intensity in untrained male wistar rats. Sci Rep.

[38] Rahimi G, Heydari S, Rahimi B, Abedpoor N, Niktab I, Safaeinejad Z, Peymani M, Seyed Forootan F, Derakhshan Z, Esfahani MHN (2021). A combination of herbal compound (sptc) along with exercise or metformin more efficiently alleviated diabetic complications through down-regulation of stress oxidative pathway upon activating nrf2-keap1 axis in age rich diet-induced type 2 diabetic mice. Nutr Metab.

[39] Razmara E, Garshasbi M (2018). Whole-exome sequencing identifies r1279x of myh6 gene to be associated with congenital heart disease. BMC Cardiovasc Disord.

[40] Ruan Y, Jin Q, Zeng J, Ren F, Xie Z, Ji K, Wu L, Wu J, Li L (2020). Grape seed proanthocyanidin extract ameliorates cardiac remodelling after myocardial infarction through pi3k/akt pathway in mice. Front pharmacol.

[41] Shao D, Di Y, Lian Z, Zhu B, Xu X, Guo D, Huang Q, Jiang C, Kong J, Shi J (2020). Grape seed proanthocyanidins suppressed macrophage foam cell formation by mirna-9 via targeting acat1 in thp-1 cells. Food & function.

[42] Sochorova L, Prusova B, Cebova M, Jurikova T, Mlcek J, Adamkova A, Nedomova S, Baron M, Sochor J (2020). Health effects of grape seed and skin extracts and their influence on biochemical markers. Mol.

[43] Valiente Alandi I, Potter SJ, Salvador AM, Schafer AE, Schips T, Carrillo Salinas F, Gibson AM, Nieman ML, Perkins C, Sargent MA (2018). Inhibiting fibronectin attenuates fibrosis and improves cardiac function in a model of heart failure. Circ.

[44] Wang H, Bei Y, Lu Y, Sun W, Liu Q, Wang Y, Cao Y, Chen P, Xiao J, Kong X (2015). Exercise prevents cardiac injury and improves mitochondrial biogenesis in advanced diabetic cardiomyopathy with pgc-1α and akt activation. Cell Physiol Biochem.

